# Cardiovascular Health, Assessed by Life’s Essential 8, Is Associated with Lower Risk of Disability Among Older, Community-Dwelling Men and Women

**DOI:** 10.3390/ejihpe15090181

**Published:** 2025-09-08

**Authors:** Xin Li, Yichen Jin, Stefania Bandinelli, Luigi Ferrucci, Toshiko Tanaka, Sameera A. Talegawkar

**Affiliations:** 1 Department of Exercise and Nutrition Sciences, Milken Institute School of Public Health, The George Washington University, Washington, DC 20052, USA; xli317@gwu.edu (X.L.); sameera.talegawkar@alumni.tufts.edu (S.A.T.); 2 Geriatric Unit, Azienda Sanitaria Firenze (ASF), 50137 Florence, Italy; stefania.bandinelli@asf.toscana.it; 3 Translational Gerontology Branch, National Institute on Aging, Baltimore, MD 21224, USA; ferruccilu@grc.nia.nih.gov (L.F.); tanakato@mail.nih.gov (T.T.)

**Keywords:** healthy aging, disability, InCHIANTI study, cardiovascular health

## Abstract

Our study examined the associations between overall cardiovascular health (CVH) and the risk of disability in older adults over 16 years of follow-up. Data from the InCHIANTI study were used and included 928 participants aged 65 years and older. Overall CVH was measured using the “Life’s Essential 8” (LE8) metric. Disability status was assessed by the Activities of Daily Living (ADL) and the Instrumental Activities of Daily Living (IADL). Higher overall LE8 score at baseline was associated with lower risks of having the first ADL among males (hazard ratio [HR] = 0.66, *p* = 0.001) and IADL disability event (HR = 0.89, *p* = 0.016) using the Cox proportional hazards model. Higher LE8 scores were also associated with ADL (odds ratio [OR] (females) = 0.80, *p* = 0.034; OR (males) = 0.55, *p* < 0.001) and IADL worsening (OR = 0.72, *p* < 0.001) using Generalized Estimating Equation models. Among older adults, better CVH assessed by the LE8 metric was associated with lower risk of ADL and IADL disabilities and their worsening over time. These findings underscore the importance of promoting CVH as a key strategy to support healthy aging and reduce disability burden. Public health interventions that target CVH components may be effective in preserving functional independence among older populations.

## 1. Introduction

With the rapid growth in the aging population, healthy aging, the continuous process of maintaining good physical and mental health, independence, as well as quality of life, (Rowe & Kahn, 1997) has emerged as an important goal in public health. Disability, commonly defined as the difficulty or lack of independence in performing activities of daily living (ADL) or instrumental activities of daily living (IADL) (Fried et al., 2004), can pose a major barrier to achieving healthy aging. Based on the data from the third wave of the European Health Interview Survey conducted in 2019, it is estimated that over half of adults aged 65 and older in Europe experience difficulty with at least one personal care or household activity (Van Eeuwijk & Angehrn, 2017). With nearly one-quarter of its population aged 65 or older, Italy ranks among the countries with the highest proportion of older adults (ISTAT, 2022; Melchiorre et al., 2022). Moreover, approximately 37% of Italians in this age group report some form of disability, with mobility-related limitations being the most prevalent (Boccardi & Mecocci, 2023). This high prevalence of disability among older adults, along with its complex associations with other chronic conditions common in this age group, can increase the risk of mortality and comorbidities and introduce increasing complexity in the provision of care within healthcare systems (Chernew et al., 2005; Fried & Guralnik, 1997). Hence, investigation of the factors associated with disability among older adults is crucial for effective public health intervention implementations.

Previous studies have demonstrated the associations between cardiovascular health (CVH) and disability among older adults (Chen et al., 2024; Cui et al., 2020; Hajjar et al., 2007; Heiland et al., 2019; Jin et al., 2019). CVH is a concept proposed and defined by the American Heart Association (AHA) initially in 2010 to identify health factors and modifiable behaviors associated with the risk of cardiovascular diseases (CVD) (Hasbani et al., 2022; Lloyd-Jones et al., 2010). “Life’s Simple 7” (LS7), a metric that encompasses 7 important predictors (smoking status, physical activity level, body mass index [BMI], diet quality, blood pressure, total cholesterol, and fasting glucose levels) was defined by the AHA to describe CVH (Lloyd-Jones et al., 2010). The LS7 metric was updated to “Life’s Essential 8” (LE8) in 2022, with the addition of sleep in the health behavior component. Sufficient and high-quality sleep is imperative to optimal health, and evidence suggests that sleep contributes additional predictive value for CVD events above the original LS7 metric (Makarem et al., 2022). Additionally, findings from a recent study suggested that older adults who reported sleep problems had greater odds of having a disability in basic activities of daily living (BADL) and IADL, compared to those who had no sleep problems (Idalino et al., 2024). This updated LE8 metric that assesses CVH via both health behaviors (diet, physical activity, nicotine exposure, sleep) and health factors (BMI, fasting glucose, cholesterol, blood pressure) can therefore serve as a robust, holistic model for comprehensively investigating the associations between overall CVH and disability risk.

The objective of this study is to investigate the associations between overall CVH, measured using the LE8 metric, and the risk of disability in older adults. We hypothesized that a higher score on the LE8 metric, indicating better CVH, is associated with a lower risk of ADL and IADL disabilities in older adults.

## 2. Methods

### 2.1. Study Design and Population

The InCHIANTI (“Invecchiare in Chianti”) study is a prospective population-based cohort conducted in Greve in the Chianti region and Bagno a Ripoli in Tuscany, Italy. Participants were enrolled from September 1998 to March 2000 via two-stage stratified sampling methods from the two cities and then followed longitudinally. Structured home interviews were conducted to collect data on study variables. Comprehensive medical and functional examinations, including the collection of blood and urine samples, were performed every three years at the study site. Detailed descriptions regarding the study design and primary objectives have been previously described (Ferrucci et al., 2000).

Data from 1453 participants, spanning from baseline to follow-up visit 4 (2013–2014), were recruited. Participants who were under 65 years old (*n* = 298), those with missing information on the components of LE8 (*n* = 216), implausible energy intake (<600 or >4000 kcal/day, *n* = 1), and diagnosed with dementia (diagnosed by experienced geriatrician and psychologists based on DSM-III-R criteria) at baseline (*n* = 10) were excluded (Supplemental Figure S1). The final analytic sample included 928 participants. For analysis of incident disability, participants who were disability-free at baseline were included, resulting in *n* = 901 for ADL and *n* = 752 for IADL. For analysis of disability progression, a total of 860 participants with available disability assessment at any follow-up visits were included for analysis. The study protocol was approved by the Institutional Review Board of the Italian National Institute of Research and Care of Aging, and written informed consent was obtained from all participants. The current secondary data analysis was reviewed and determined to be exempt by the Institutional Review Board of the National Institutes of Health.

### 2.2. Derivation of the LE8 Metric

Each component within the LE8 metric is assigned an ordinal point system ranging from 0 to 100 points, with context-specific cutoffs implemented to better capture inter-individual variations and CVH improvements over time (Lloyd-Jones et al., 2022). The overall score of the LE8 metric is calculated by taking the average of the scores from all eight individual components that make up the LE8. A score of 0 to 49 is considered “Low LE8” (indicating low CVH), 50 to 79 is considered “Moderate LE8” (indicating moderate CVH), and 80 to 100 is “High LE8” (indicating high CVH) (Lloyd-Jones et al., 2022). In our analyses, due to the small number of participants falling into the high LE8 category (*n* = 9), we combined the moderate and high LE8 categories.

The scoring criteria for each LE8 component are displayed in Supplemental Table S1. Within the InCHIANTI study, a validated food frequency questionnaire developed for the Italy site of the European Prospective Investigation on Cancer and Nutrition was used to collect self-reported dietary intake data (Bartali et al., 2004). The Mellen’s score, ranging from zero to nine, was used to assess diet quality based on adherence to the Dietary Approaches to Stop Hypertension dietary pattern.

For physical activity, self-reported physical activity level for the past year was collected. Smoking status was determined based on cigarette use only because the information on secondhand smoke exposure and inhaled nicotine delivery system use was unavailable in the cohort. Self-reported sleep duration in the past month was obtained via a standard questionnaire.

The BMI (kg/m^2^) of the participants was calculated using measured weight (kg) and height (m). Blood pressure was measured in a supine position using a mercury sphygmomanometer, with a 2 min interval between each of the three readings (Ferrucci et al., 2000; Jin et al., 2019). Assessments were performed at the study clinic visit. Information on medication use relating to blood pressure was obtained through home interviews.

Total and HDL cholesterol and fasting blood glucose were measured through serum samples collected from participants who had fasted for at least 8 h. Detailed information on the assessment assays has been described previously (Jin et al., 2019). Non-HDL cholesterol was calculated as subtracting HDL from total cholesterol and used for score derivation. Hemoglobin A1c (HbA1c) levels were used to estimate the blood glucose profile based on the LE8 criteria, but no HbA1c data were collected in the InCHIANTI study. We instead used average fasting glucose levels and a conversion chart from the American Diabetes Association to calculate the scores for participants (https://professional.diabetes.org/diapro/glucose_calc, accessed on 16 May 2023). This is based on a formula of HbA1c = (fasting glucose [mg/dL] + 46.7)/28.7. Information on medication use relating to blood lipids and diabetes was obtained through home interviews.

### 2.3. Assessment of Disability

The disability status of participants in the InCHIANTI study was assessed at baseline and each follow-up visit by evaluating their ability to perform ADL and IADL (Katz et al., 1963; Lawton & Brody, 1969). The ADL questionnaire includes six functional tasks: bathing, dressing, toileting, getting into and out of bed, continence, and eating. The IADL questionnaire includes eight tasks, including using the telephone, shopping, food preparation, doing light housework, laundering, transportation, taking medications, and handling finances. The participants were classified as having a disability if the number of ADL and IADL they were unable to perform was greater than zero.

The progression of both ADL and IADL disabilities over time was determined by comparing the number of disabilities reported during each follow-up visit to the baseline disability status. If the number of ADL and IADL disabilities increased, this was deemed indicative of worsening disability status. The worsening of ADL and IADL disability was analyzed as a dichotomous outcome.

### 2.4. Covariate

Covariates were determined based on univariate analyses or previous literature on disability and included age, sex, study site, years of education, cognitive status, depression, and number of chronic diseases. The Mini-Mental State Examination (MMSE) was used to assess participants’ cognitive status, and an MMSE score lower than 24 was an indicator of impaired cognition (Dick et al., 1984). The depression status of participants was assessed via the Center for Epidemiological Studies-Depression (CES-D) scale. A CES-D score of 16 or above was indicative of depression (Milaneschi et al., 2011). Chronic diseases including cancer, heart failure, coronary heart disease, stroke, chronic lung disease, hip arthritis, liver disease, gastrointestinal disease, peripheral arterial disease, Parkinson’s disease, and renal disease, were assessed based on the criteria used in the Women’s Health and Aging Study (Lafferty et al., 1996).

### 2.5. Statistical Analysis

Baseline sociodemographic characteristics and health status variables were reported as mean (standard deviation [SD]) or percentage. The differences in baseline characteristics between Moderate/High LE8 and Low LE8 scores were tested using a *t*-test and chi-square test for continuous and categorical variables, respectively. Incidence rates of ADL and IADL were calculated per 1000 person-years. Cox proportional hazard models were used to examine the hazard ratio of first ADL and IADL disability by baseline LE8 score and its components among participants who reported no disability at baseline. The assumption of proportional hazards was verified, and the concordance index was used to evaluate model fit. Generalized estimating equations (GEE) models with unstructured correlation were used to examine the associations between baseline LE8 score and its components with ADL and IADL progression over 16 years of follow-up, adjusting for baseline covariates and time. Effect modification by time variables was examined, and there were no statistically significant associations for both ADL and IADL. Model accuracy was evaluated using the Brier score, accounting for intrapersonal correlation. For all analyses, modification by sex was also evaluated by including the interaction term between LE8 and sex. When the interaction term was significant (*p* < 0.05), results from stratified analyses were presented.

For sensitivity analyses, we assessed the competing risk of death by calculating cumulative incidences. The Fine-Gray model was then used to examine the subdistribution hazard ratios (SHRs) of disability accounting for death events. We also examined the associations between LE8 score and its components with ADL and IADL progression among participants who were disability-free at baseline. To address potential bias due to missing data, we applied stabilized Inverse Probability Weighting (IPW) for the associations between LE8 and disability progression. The stabilized IPW was calculated as a ratio of the marginal probability of not being excluded to the conditional probability from the logistic regression model of not being excluded on covariates. These weights were then applied in the GEE models, allowing us to estimate associations while accounting for potential confounding and selection bias due to missingness. Model diagnostics, including proportional subdistribution hazard checks, were conducted. All analyses were performed using R version 4.2.3, and a two-tailed alpha level of 0.05 was used to determine statistical significance.

## 3. Results

The study sample was selected from the 1155 participants over the age of 65 at baseline, representing 79.5% of the study (Supplemental Figure S1). The analytical sample consisted of 928 participants (80.3%) with data on LE8, diet, and free of prevalent dementia. Compared to participants in the analytic sample, those who were excluded were older (81.6 years old vs. 73.9 years old), higher percentage of female participants (63.4% vs. 55.1%), and more likely to report ADL (39.2% vs. 2.9%) or IADL (65.6% vs. 19.0%) disability (Supplemental Table S2).

### 3.1. Overall LE8 Distribution by Sex

**Figure 1:**
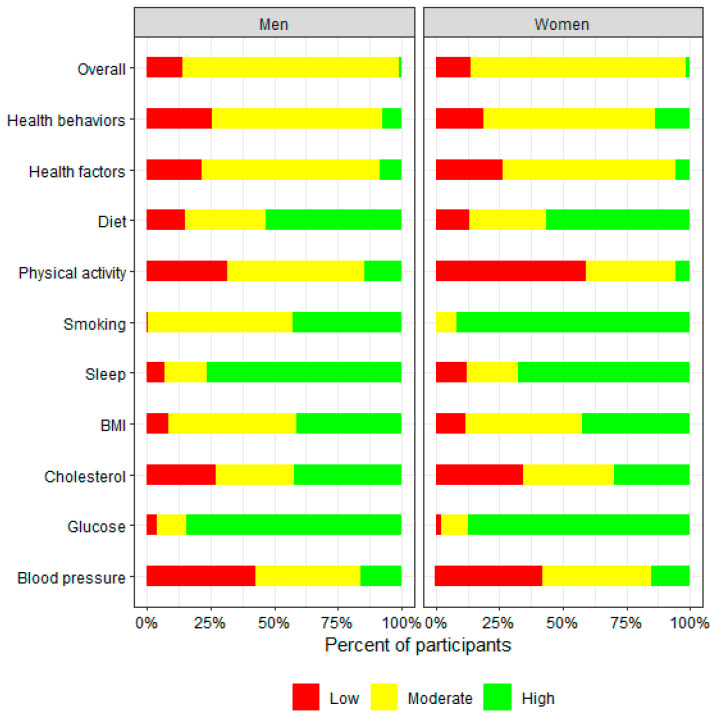
Sex-stratified distribution of overall LE8 score and individual LE8 component scores at baseline among InCHIANTI study participants aged 65 and older. Score range for “Low LE8” is 0–49; for “Moderate LE8” is 50–79; for “High LE8” is 80–100.

The sex-stratified distribution of the overall LE8 score and scores of individual components is presented in Figure 1. In general, both male and female participants had similar overall LE8 scores, health behavior scores, and health factor scores. Scores for diet, BMI, cholesterol, glucose, and blood pressure components of the LE8 were comparable. While a significant proportion of participants of both sexes were identified as having low levels of physical activity at baseline, a greater percentage of female participants had lower habitual physical activity levels compared with the male participants. Compared with female participants, more male participants were classified as having higher sleep scores, suggesting sufficient sleep duration. More female participants were categorized with higher scores for smoking status, indicating a larger proportion had never smoked compared with their male counterparts.

The baseline sociodemographic and health characteristics are described in Table 1. The mean age (SD) of the cohort was 74 (6.6) years, with 55% being female. Among the 928 participants, approximately 82% were classified as having Moderate/High LE8 at baseline. No significant differences in age, years of education, and cognitive status were observed among participants with Low LE8 and those with Moderate/High LE8 at baseline. Participants with Low LE8 exhibited a higher prevalence of depression and at least one chronic disease at baseline compared to those who were in Moderate/High LE8. Twenty-seven and 176 participants reported having ADL and IADL disabilities, respectively. Participants in the Low baseline LE8 also had a greater prevalence of ADL or IADL disability.

### 3.2. Baseline CVH and Risks of Developing ADL/IADL Disabilities

During 16 years of follow-up, 247 ADL (82 males and 165 females) and 446 IADL cases developed, and the incidence rates are 24.8 and 65.0 per 1000 person-years for ADL and IADL, respectively. The associations between baseline CVH and the risk of developing ADL or IADL disability over 16 years of follow-up are presented in Table 2. The association between LE8 and incident ADL disability was modified by sex (*p* for interaction < 0.05). In female participants, no statistically significant associations were observed. However, among males, a 1 SD increment in the overall LE8 score was associated with a 34% (HR: 0.66, 95% CI: 0.52–0.85) reduction in the risk of developing ADL disability. Male participants in the Moderate/High LE8 group also had a lower risk of developing ADL disability compared to those in the Low LE8 group (HR: 0.44, 95%CI: 0.25–0.77). In female participants, the LE8 health behavior score was associated with a reduced risk of developing ADL disability (HR: 0.82, 95% CI: 0.68–0.97), while in male participants, the LE8 health factor score was associated with reduced ADL disability risk (HR: 0.76, 95% CI: 0.60–0.96). Analyses of individual components demonstrated that higher scores on baseline physical activity were associated with a reduced risk of developing ADL disability in both sexes. In males, a better baseline blood pressure profile was associated with a reduced risk of developing ADL disability (HR: 0.70, 95% CI: 0.54–0.91). Contrary to our expectations, female participants who received higher scores for sleep duration had a higher risk of developing ADL (HR: 1.22, 95% CI: 1.03–1.43). After accounting for the competing risk of death, a higher LE8 score was associated with a lower risk of ADL disability (SHR: 0.87, 95% CI: 0.77–0.99) (Supplemental Table S3).

Unlike what was observed for ADL, the associations between LE8 and incident IADL disabilities were not modified by sex (Table 2). On both a continuous (HR: 0.89, 95% CI: 0.80–0.98) and categorical scale (HR: 0.72, 95% CI: 0.56–0.92), higher LE8 scores were associated with a reduced risk of incident IADL over a 16-year follow-up. Additionally, per 1 SD increment in the LE8 health behavior score was associated with a significant 13% reduction in the risk of IADL disability. Among the four health behavior components, a higher level of baseline physical activity was particularly associated with a lower risk of IADL disability, with a hazard ratio of 0.78 (95% CI: 0.70–0.87).

### 3.3. Baseline CVH and Risks of ADL/IADL Disabilities Progression

Higher baseline LE8 scores were associated with lower risk of ADL and IADL progression (Table 3). Similar to what we observed for the incidence of ADL disability, the longitudinal associations between LE8 and its components with ADL worsening were modified by sex. Higher LE8 health behavior scores were associated with reduced risks of both ADL and IADL worsening in both sexes, whereas higher LE8 health factor scores were associated with lower risks of IADL and ADL worsening in male participants only. Higher physical activity and better blood pressure at baseline were associated with reduced risk of IADL and ADL (specifically for male participants) disability progression. In addition, higher scores on BMI were associated with a lower risk of IADL worsening (OR: 0.86, 95% CI: 0.76–0.97). Comparable results were observed in the sensitivity analyses on the associations between LE8 with ADL and IADL disability worsening among participants without disability at baseline, but with larger effect sizes (Supplemental Table S4). When using the model weighted by stabilized IPW, higher LE8 was also associated with a lower risk of both ADL and IADL worsening, but the associations were attenuated for the risk of ADL worsening in female participants (Supplemental Table S5).

## 4. Discussion

In a community-based cohort of older Italians, better CVH, indicated by a higher overall LE8 score at baseline, was associated with a lower risk of developing ADL and IADL disability, as well as worsening over a 16-year follow-up. Interestingly, we observed sex-specific associations only between LE8 health behaviors and health factors with ADL disability. Female participants with higher LE8 health behavior scores and male participants with higher LE8 health factor scores demonstrated a lower risk of ADL disability over time, suggesting potential factors that protect against age-associated disability differ by sex.

Physical function is an important element of healthy aging. Physical function preservation is crucial to managing self-care tasks and engaging in social activities, which in turn will impact the overall physical and mental health status of older adults. The findings of the current analysis demonstrate protective associations between CVH and the risks of developing disability and its progression in older adults. These findings were consistent with previous research that examined the associations between overall CVH and age-related physical function decline or disabilities (Devulapalli et al., 2016; García-Hermoso et al., 2017; Jin et al., 2019). In the InCHIANTI study, per one-point increase in the overall LS7 score was associated with 23% and 17% lower odds of ADL (*p* < 0.001) and IADL (*p* < 0.001) disability, as well as slowing further decline over 9 years (Heiland et al., 2019). Comparable findings were reported in the National Health and Nutrition Examination Survey (NHANES). Using data from roughly 4000 participants across four survey cycles (2005–2006, 2007–2008, 2009–2010, and 2011–2012), Devulapalli et al. found that per one-point increase in the overall CVH score was associated with 10% reduced odds of ADL disability (Devulapalli et al., 2016). A similar study in 2024 examined the health impacts of CVH on osteoarthritis and disability among adults aged 20 years and older in the NHANES using the LE8 metric showed that better CVH was associated with a lower prevalence of disability in various domains compared to low CVH, which is consistent with our findings (Chen et al., 2024).

Our findings from the primary analyses suggested a significant positive association between ideal sleep duration (defined by the LE8 metric as sleeping between 7 and 9 h) and increased risk of ADL disability among females. However, this association was attenuated in our sensitivity analyses using inverse probability weighting. Previous studies have examined the influence of sleep duration on the development of disability, suggesting that both short and prolonged sleep duration are associated with an increased risk of disability in older adults (Nakakubo et al., 2016; Zhang et al., 2019). However, most of these studies have focused on the independent effect of sleep on disability and have not considered the potential interactions between sleep and other CVH factors (Bock et al., 2022; Dashti et al., 2015; Grandner et al., 2016). This is noteworthy considering the strong associations between sleep, disability, and the likelihood of concurrent disability and CVD events in the aging population (Plichart et al., 2010; Wilby, 2019).

In general, our findings suggest that CVH behaviors were particularly important in reducing disability among females, whereas CVH factors appeared to have a more prominent role in males. This observed sex-specific difference in CVH pathways related to disability prevention can be attributed to various factors, including physiological differences, social and environmental factors that shape an individual’s lifestyle choices or health-seeking behaviors. For example, there are notable differences in the habitual physical activity levels between these two sexes, with older females tending to be more sedentary or less likely to participate in moderate or vigorous physical activity compared to males of the same age (Lee, 2005). An improvement in physical activity levels among female participants over time may yield greater health benefits to prevent ADL disability compared to male participants who already have a higher baseline physical activity level. While males have a higher risk of developing hypertension at earlier stages of life (Burt et al., 1995; Khoury et al., 1992; Wiinberg et al., 1995), they may already exhibit pre-hypertensive symptoms during midlife, such as excessive sodium intake (Heller et al., 2023). The sustained sub-optimal blood pressure profiles at midlife and subsequent increased risk of hypertension due to poor disease management among male participants may contribute significantly to an elevated risk of ADL disability observed in late life.

Similarly, blood glucose and BMI were also found to be associated with reduced risks of disability, especially for IADL disability in both sexes. Blood pressure, blood glucose, and BMI are three health factors closely interrelated and potentially amplifying their impact on functional outcomes. Several studies have suggested that being overweight, compared to being underweight or obese, may offer protective effects against disability (both ADL and IADL) and mortality in older adults (Borda et al., 2021; Corona et al., 2014; Imai et al., 2008). Even among those already living with disability, individuals with higher BMI (≥27.5 kg/m^2^) have demonstrated longer survival compared to those with BMI < 18.5 kg/m^2^-possibly reflecting better overall nutritional status. Supporting this, a community-based study in Shanghai, China found that underweight older adults with hypertension had a 65% higher odd of developing IADL disability (Su et al., 2017). It is important to note that IADL decline often represents a transitional stage in functional loss-one that may still be reversible before progressing to ADL disability. This makes it a critical window for intervention. Early identification and management of CVH factors through sustainable strategies, like promoting a nutrient-dense, balanced diet, can play a key role in preserving physical function and supporting healthy aging.

While we observed sex differences in the association between LE8 health behavior and ADL disability, these differences were largely driven by the unexpected associations with sleep. For all other analyses, the direction and magnitude of risk reduction for LE8 health behavior and health factor scores were similar across sexes. Most individual LE8 components were not independently associated with incident disability and disability worsening. Our findings reinforce the idea that the cumulative effect of overall CVH plays a more substantial role in physical function preservation in older adults. Among all the components, physical activity had the greatest effect on the risk of ADL/IADL disability and progression, supporting the importance of promoting an active lifestyle as a key strategy for delaying or preventing disability in older adults.

Several limitations need to be noted in our study. First, the single measurement of the LE8 score at baseline may limit causal inferences and not capture the entire trajectory of CVH over time, which may offer valuable information for disability risk prediction. Also, changes in covariates over time were not accounted for in our models because of data availability, which could potentially introduce bias. Second, the information on health behaviors and disability assessment was collected via a self-reported standard questionnaire, which is subject to recall bias and potentially less accurate information due to social desirability, potentially resulting in measurement error and misclassification. The conversion of HbA1c to estimated blood glucose cutoffs, which was used for score calculation, may also introduce misclassification. While not considered a limitation, it is important to acknowledge that we modified the LE8 scores for diet quality, physical activity, and smoking status from the originally specified LE8 metric by the AHA. The original LE8 guideline suggested standardizing to NHANES-based thresholds to calculate the diet score categories. However, considering that the participants in our analysis are older adults from rural Italy, it would be more practical to utilize the cut-off values derived from the InCHIANTI study to better capture the actual diet quality of these individuals, rather than relying on the NHANES (generalizable to the American population)-based cut-offs. Participants excluded from the study due to missing data on LE8 and covariates were older and had a higher prevalence of ADL and IADL disability, reflecting the robustness of the participants in the analytical sample. Because the most vulnerable individuals were excluded, the effect of CVH on disability could be either overestimated if those excluded are less responsive to lifestyle change or underestimated if lifestyle changes are particularly beneficial for those who are prone to disabilities. This could limit the generalizability of study results to populations that are older and more vulnerable to ADL/IADL disability. Further, this difference in analytical sample may introduce selection bias; however, we performed sensitivity analysis using the IPW model to address the potential bias introduced by these exclusions. While the study adjusted for several key confounding factors, there could be residual confounding by factors that were not measured in this study, including unmeasured health conditions. Our study has several strengths. It is a prospective longitudinal cohort of older adults with a long follow-up time of 16 years. Although self-reported, data on health behaviors were collected using a validated standardized questionnaire, and data on health factors were collected via blood tests. Available information on the two validated and well-documented measures of self-reported functional status, ADL and IADL, at both baseline and over time, allowed us to capture the decline of physical function of these participants over time, which is also another significant strength. Information on several confounders and covariates, including cognitive impairment and depression, was also available in the cohort. In addition, our study had an adequate sample size, meeting the commonly recommended threshold of more than 10 events per variable.

Recognizing the growing burden of chronic disease and functional decline in an aging population, Italy has implemented several national policies and health programs aimed at promoting CVH among older adults. Notably, the “Progetto Cuore” (Heart Project), initiated by the Instituto Superiore di Sanità in 1998, serves as a cornerstone surveillance program for CVD risk factors in the adult population (Panico et al., 2008). More recently, Italy’s National Cardiovascular and Cerebrovascular Diseases Plan has introduced structured screening initiatives targeting individuals aged 70 and above, aiming to detect and manage CVD risk early through general practitioner-led outreach (EU SHD Coalition, 2024). Complementary programs such as the National Prevention Plan (2020–2025) focus on lifestyle promotion and cardiovascular risk monitoring, including tailored approaches for older adults (Alonzo et al., 2023; Barbabella et al., 2022). Despite these comprehensive efforts, challenges remain in optimizing CVH management and using these existing resources effectively to preserve physical function in older adults in Italy. Our findings may offer insights into the possible connections between CVH and physical function, thereby helping to raise awareness of these associations and guide the risk assessments used in these screening programs to identify older adults who may benefit the most from which type of targeted interventions.

## 5. Conclusions

Better overall CVH assessed by the LE8 metric is associated with lower risks of ADL and IADL disabilities development over time among older adults from the InCHIANTI study. Both health behaviors and health factors contribute equally to staving off the development of ADL and IADL disability. The findings of this study underscore the importance of implementing a public health strategy to address the health and behavioral risk factors for physical function preservation and thus healthy aging promotion.

## Figures and Tables

**Table 1:** Baseline sociodemographic and health characteristics (mean [standard deviation] or percentage) by the category of overall LE8 score among InCHIANTI study participants aged 65 years and older.

		Cardiovascular Health Score		
Variables	Total	Low LE8	Moderate/High LE8	*p*-Value ^1^
n	928	168	760	
Age (years)	73.9 (6.6)	73.6 (6.3)	74.0 (6.7)	0.435
Sex, females (%)	511 (55.1)	92 (54.8)	419 (55.1)	0.999
Study Site, Bagno a Ripoli (%)	486 (52.4)	83 (49.4)	403 (53.0)	0.444
Education (years)	5.5 (3.2)	5.5 (3.2)	5.5 (3.2)	0.877
Cognitive Impairment ^2^ (%)	220 (23.7)	36 (21.4)	184 (24.2)	0.505
Depression (%)	186 (20.0)	46 (27.4)	140 (18.4)	0.012
Presence of Chronic Disease ^3^ (%)	573 (61.7)	118 (70.2)	455 (59.9)	0.016
ADL Disability (%)	27 (2.9)	13 (7.7)	14 (1.8)	<0.001
IADL Disability (%)	176 (19.0)	43 (25.6)	133 (17.5)	0.021

^1^ *t*-test and chi-square test were used for continuous and categorical variables, respectively. ^2^ Baseline cognitive impairment was indicated by an MMSE score lower than 24. ^3^ Chronic disease included cancer, heart failure, coronary heart disease, stroke, chronic lung disease, hip arthritis, liver disease, gastrointestinal disease, peripheral arterial disease, Parkinson’s disease, and renal disease.

**Table 2:** Multivariable-adjusted Hazard Ratios of ADL and IADL Disability by Overall LE8 Score (Per 1 SD) and Individual LE8 Component ^1^.

LE8 Score or Components		Hazard Ratios (95% CI)				
		ADL Disability (Sex-Stratified) ^2^		*p*-Value	IADL Disability	*p*-Value
Overall LE8 Score ^3^		F	0.95 (0.81, 1.12)	0.571	0.89 (0.80, 0.98)	0.016
		M	0.66 (0.52, 0.85)	0.001		
Moderate/High vs. Low		F	0.74 (0.50, 1.08)	0.120	0.72 (0.56, 0.92)	0.009
		M	0.44 (0.25, 0.77)	0.004		
Health Behavior Score		F	0.82 (0.68, 0.97)	0.025	0.87 (0.79, 0.97)	0.009
		M	0.81 (0.65, 1.01)	0.067		
Health Factor Score		F	0.97 (0.83, 1.14)	0.738	0.97 (0.88, 1.06)	0.526
		M	0.76 (0.60, 0.96)	0.021		
Health Behaviors	Diet	F	0.89 (0.76, 1.06)	0.188	0.96 (0.87, 1.06)	0.419
		M	0.98 (0.77, 1.26)	0.890		
	Physical Activity	F	0.72 (0.60, 0.87)	0.001	0.78 (0.70, 0.87)	<0.001
		M	0.65 (0.51, 0.84)	0.001		
	Smoking	F	0.99 (0.79, 1.25)	0.955	0.96 (0.87, 1.07)	0.466
		M	0.90 (0.72, 1.12)	0.330		
	Sleep	F	1.22 (1.03, 1.43)	0.018	0.94 (0.85, 1.04)	0.217
		M	0.86 (0.68, 1.10)	0.238		
Health Factors	BMI	F	1.03 (0.89, 1.19)	0.701	1.01 (0.92, 1.11)	0.832
		M	0.95 (0.75, 1.21)	0.673		
	Cholesterol	F	1.05 (0.89, 1.24)	0.572	1.00 (0.91, 1.10)	0.960
		M	0.87 (0.69, 1.09)	0.223		
	Blood Glucose	F	0.89 (0.76, 1.04)	0.146	0.97 (0.88, 1.07)	0.535
		M	0.93 (0.75, 1.15)	0.489		
	Blood Pressure	F	0.95 (0.81, 1.13)	0.592	0.94 (0.85, 1.04)	0.231
		M	0.70 (0.54, 0.91)	0.007		

^1^ This table presents the hazard ratios [HRs] (95% confidence intervals) from the Cox proportional hazard models that adjusted for participants’ age, sex (for IADL analyses), study site, years of education, baseline cognitive status, baseline depression status, presence of chronic disease at baseline. ^2^ Sex was shown to be an effect modifier for the association between overall LE8 scores and its 8 components and risk of ADL disability only. Hence, sex-stratified HRs for ADL disability were reported. ^3^ The concordance index for the associations between the overall LE8 score with incident ADL in males and females and IADL were 0.74, 0.81, and 0.72, respectively.

**Table 3:** Multivariable-adjusted Odds Ratios of ADL and IADL Progression by Overall LE8 Score (Per 1 SD) and Individual LE8 Component ^1^.

LE8 Score or Components		Odds Ratios (95% CI)				
		ADL Disability (Sex-Stratified) ^2^		*p*-Value	IADL Disability	*p*-Value
Overall LE8 Score ^3^		F	0.80 (0.64, 0.98)	0.034	0.72 (0.63, 0.83)	<0.001
		M	0.55 (0.41, 0.74)	<0.001		
Moderate/High vs. Low		F	0.46 (0.27, 0.76)	0.003	0.40 (0. 28, 0. 56)	<0.001
		M	0.30 (0.16, 0.57)	<0.001		
Health Behavior Score		F	0.77 (0.61, 0.96)	0.019	0.81 (0.71, 0.92)	0.001
		M	0.74 (0.59, 0.94)	0.011		
Health Factor Score		F	0.83 (0.67, 1.04)	0.103	0.81 (0.71, 0.92)	0.001
		M	0.68 (0.51, 0.90)	0.007		
Health Behaviors	Diet	F	0.91 (0.73, 1.12)	0.368	0.99 (0.88, 1.12)	0.903
		M	0.99 (0.77, 1.28)	0.950		
	Physical Activity	F	0.56 (0.43, 0.73)	<0.001	0.56 (0.49, 0.65)	<0.001
		M	0.43 (0.32, 0.59)	<0.001		
	Smoking	F	0.98 (0.76, 1.27)	0.887	0.95 (0.83, 1.08)	0.430
		M	0.93 (0.73, 1.19)	0.582		
	Sleep	F	1.10 (0.91, 1.33)	0.340	0.89 (0.79, 1.01)	0.071
		M	0.81 (0.63, 1.05)	0.109		
Health Factors	BMI	F	0.88 (0.72, 1.09)	0.250	0.86 (0.76, 0.97)	0.015
		M	0.90 (0.69, 1.19)	0.466		
	Cholesterol	F	1.01 (0.81, 1.26)	0.940	1.01 (0.89, 1.14)	0.898
		M	0.89 (0.68, 1.17)	0.403		
	Blood Glucose	F	0.88 (0.70, 1.10)	0.255	0.85 (0.75, 0.97)	0.014
		M	0.81 (0.63, 1.04)	0.103		
	Blood Pressure	F	0.80 (0.63, 1.01)	0.065	0.82 (0.72, 0.93)	0.002
		M	0.57 (0.39, 0.83)	0.003		

^1^ This table presents the odds ratios [ORs] (95% confidence intervals) from the GEE models with unstructured covariance that adjusted for participants’ age, sex, study site, years of education, baseline cognitive status, baseline depression status, presence of chronic disease at baseline. ^2^ Sex was shown to be an effect modifier for the association between overall LE8 scores and its 8 components and risk of ADL disability only. Hence, sex-stratified ORs for ADL disability were reported. ^3^ The Brier scores for the associations between the overall LE8 score with ADL in males and females and IADL progression were 0.10, 0.11, and 0.16, respectively.

## Data Availability

Data described in the manuscript, code book, and analytic code will be made available upon request pending an application to and approval by the InChianti Study cohort investigators and research team at the NIA (https://www.nia.nih.gov/inchianti-study, accessed on 30 April 2023).

## Supplementary Materials

The following supporting information can be downloaded at: https://www.mdpi.com/article/10.3390/ejihpe15090181/s1, Figure S1: Participant flow diagram for current analysis; Table S1: Modified LE8 scoring criteria used for current analysis; Table S2: Comparisons of covariates between participants being excluded and included in the analysis; Table S3: Cumulative risks and sub-distribution hazard ratios (SHRs) of disability per 1 standard deviation (SD) increment of overall LE8 score accounting for competing risk of death from the Fine-Gray models; Table S4: Multivariable-adjusted odds ratios of ADL and IADL progression by overall LE8 score and individual LE8 component among disability-free participants at baseline; Table S5: Multivariable-adjusted odds ratios of ADL and IADL progression by overall LE8 score (per 1 SD) with inverse probability weights (IPW).

## Abbreviations

The following abbreviations are used in this manuscript:

ADL	Activities of daily living
CVH	Cardiovascular health
HRs	Hazard ratios
IADL	Instrumental activities of daily living
LE8	Life’s Essential 8
LS7	Life’s Simple 7
ORs	Odds ratios
SD	Standard deviation
