# Supplementary material for: Cardiovascular Health, Assessed by Life’s Essential 8, Is Associated with Lower Risk of Disability Among Older, Community-Dwelling Men and Women
Source: Eur J Investig Health Psychol Educ. 2025 Sep 8;15(9):181. doi: 10.3390/ejihpe15090181 (PMC12468947; doi:10.3390/ejihpe15090181)
Supplement: Supplementary file 1 [file ejihpe-15-00181-s001.zip › ejihpe-3649505-supplementary.pdf]

Supplemental Figure S1. Participant flow diagram for current analysis

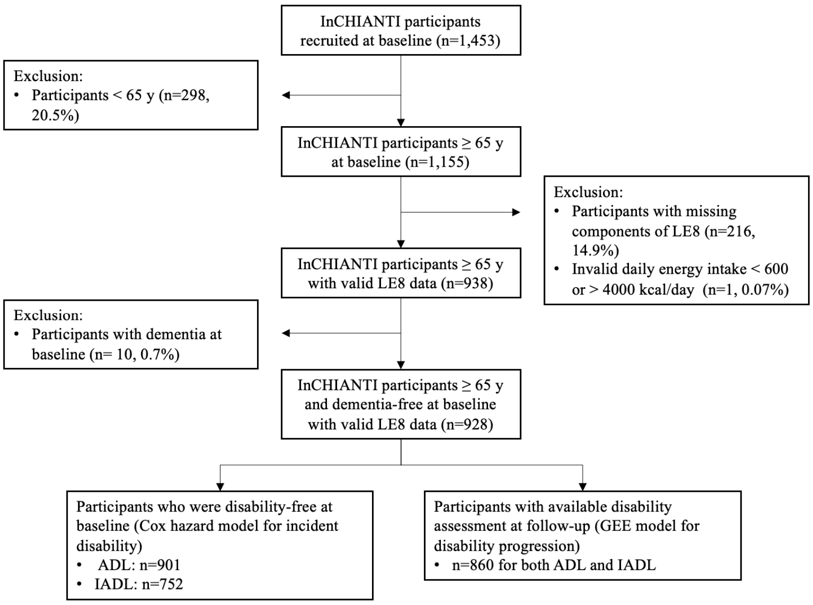

Supplemental Table S1. Modified LE8 Scoring Criteria Used for Current Analysis[1]

Domain	CVH metric	Method of measurement	Quantification of CVH metric used in current analysis
Health Behaviors	Diet[2]	Self-reported via Food Frequency Questionnaire (FFQ)	Quantiles of DASH-style diet adherence (Mellen's score[3])<br><br><u>Points</u> <u>Quantile</u><br>100 ≥95th percentile (ideal diet)<br>80 75th- 94th percentile<br>50 50th-74th percentile<br>25 25th-49th percentile<br>0 1st-24th percentile<br>(bottom/least ideal diet)
	Physical Activity	Self-reported moderate or vigorous activity time via standard questionnaire	Metrics: Moderate or vigorous activity time (hrs/wk)<br><br><u>Points</u> <u>Time</u><br>100 Walks >5km/day for > 5 days/wk<br>90 Intense exercise many times/wk<br>80 Moderate exercise > 3 hrs/week<br>60 Moderate 1-2 hrs or light > 4 hrs/ wk<br>40 Light exercise 2-4 hrs/week<br>20 Mostly sitting/some walking<br>0 Hardly any physical activity
	Smoking status	Self-reported smoking status via standard questionnaire	Metric: Cigarette use status<br><br><u>Points</u> <u>Status</u><br>100 Never smokers<br>75 Former smokers quit > 5 yrs<br>50 Former smokers quit >1 but ≤5 yrs<br>25 Former smokers quit <1yr<br>0 Current smokers
	Sleep Health	Self-reported sleep duration via standard questionnaire	Metric: Average hours of sleep per night<br><br><u>Points</u> <u>Average Hours of Sleep per Night</u><br>100 7 - <9<br>90 9 - < 10<br>70 6 - < 7<br>40 5 - < 6 or ≥ 10<br>20 4 - < 5<br>0 < 4
Health Factors	BMI	Measured weights(kilograms) and heights (meters)	Metric: BMI (kg/m^2)<br><br><u>Points</u> <u>Level</u><br>100 <25<br>70 25.0-29.9<br>30 30.0- 34.9<br>15 35.0- 39.9<br>0 ≥40.0
	Blood Lipids	Total and HDL cholesterol measured through blood tests used to	Metric: Non-HDL cholesterol (mg/dL)<br><br><u>Points</u> <u>Level</u><br>100 <130

		calculate non-HDL cholesterol	60 ≥ 130 and < 160<br>40 ≥ 160 and < 190<br>20 ≥ 190 and < 220<br>0 ≥ 220<br>*Participants with current lipid-lowering medication use are deducted for an additional 20 points.
	Blood Glucose	Fasting glucose level from serum samples[4]	Metrics: Fasting glucose level (mg/dL) and HbA1c (%)<br><br><u>Points</u> <u>Level (FBG, HbA1c)</u><br>100 FBG < 100 mg/dL<br>60 FBG ≥ 100 mg/dL and < 126 mg/dL<br>40 FBG ≥ 126 mg/dL and < 154 mg/dL<br>HbA1c < 7%<br>30 FBG ≥ 154 mg/dL and < 183 mg/dL<br>HbA1c ≥ 7-7.9%<br>20 FBG ≥ 183 mg/dL and < 212 mg/dL<br>HbA1c ≥ 8-8.9%<br>10 FBG ≥ 212 mg/dL and < 240 mg/dL<br>HbA1c ≥ 9-9.9%<br>0 FBG ≥ 240 mg/dL or HbA1c ≥ 10.0%<br>*Participants with current antihyperglycemic medication use are deducted for an additional 20 points until 0.
	Blood Pressure	Systolic blood pressure (SBP) and diastolic blood pressure (DBP) measured by mercury sphygmomanometer in supine position	Metrics: SBP and DBP value (mmHg)<br><br><u>Points</u> <u>Level (SBP, DBP)</u><br>100 <120 and < 80 (optimal)<br>75 120-130 and < 80<br>50 130-140 and <90<br>25 140-160 and <100<br>0 ≥ 160 and/or ≥ 100<br>*Participants with current hypertensive medication use are deducted for an additional 20 points.

[1]This LE8 scoring criteria for current analysis is referenced on the metrics for measurement and quantitative assessment of CVH published by the AHA in 2022(Donald M. Lloyd-Jones et al., 2022) and modified based on available information for each component.

[2] The original LE8 metric recommends the usage of the most recent or relevant NHANES data to quintile the diet score. But we used the data-driven cut-offs from the InCHIANTI study in the current analysis, as the study population is from Italy.

[3] The Mellen's DASH score, ranging from 0 to 9, was used to assess participants' consumption of nine nutrients (protein, fiber, magnesium, calcium, potassium, total fat, saturated fat, total cholesterol, sodium) and adherence to DASH diet in the current analysis. Quantification of diet in the current analysis is based on the quantile distribution of participants' Mellen's score. For example, participants with Mellen's score in the 1st-24th percentile are assigned 0 points, and those with Mellen's score in the 50th- 74th are assigned 50 points.

[4] HbA1c data were not collected in the InCHIANTI study. We used the average fasting glucose level of participants and converted the values to HbA1c using the conversion chart from the American Diabetes Association.

Supplemental Table S2. Comparisons of covariates between participants being excluded and included in the analysis

Variables	Excluded	Included for analysis	p-value[1]
n	227	928	
Age (years)	81.6 (8.5)	73.9 (6.6)	<0.001
Sex, females (%)	144 (63.4)	511 (55.1)	0.027
Education (years)	13.1 (93.2)	5.5 (3.2)	0.013
Cognitive Impairment[2] (%)	149 (65.6)	220 (23.7)	<0.001
Depression (%)	55 (36.4)	186 (20.0)	<0.001
Presence of Chronic Disease[3] (%)	98 (43.2)	573 (61.7)	<0.001
ADL Disability (%)	89 (39.2)	27 (2.9)	<0.001
IADL Disability (%)	149 (65.6)	176 (19.0)	<0.001

[1] T-test and chi-square test were used for continuous and categorical variables, respectively

[2] Baseline cognitive impairment was indicated by an MMSE score lower than 24

[3] Chronic disease included cancer, heart failure, coronary heart disease, stroke, chronic lung disease, hip arthritis, liver disease, gastrointestinal disease, peripheral arterial disease, Parkinson's disease, and renal disease

Supplemental Table S3. Cumulative Risks and Subdistribution Hazard Ratios (SHRs) of Disability per 1 Standard Deviation (SD) Increment of Overall LE8 Score Accounting for Competing Risk of Death from the Fine-Gray models[1]

Outcomes	10-year cumulative incidence[1]			
	Disability	Death	SHR (95% CI)[2]	p-value
ADL	16.9%	22.5%	0.87 (0.77, 0.99)	0.037
IADL	44.0%	14.1%	0.91 (0.81, 1.01)	0.067

[1] Analyses presented were restricted to participants who were free of ADL or IADL disability at baseline.

[2] Cumulative incidences for disability and death were calculated over a 10-year follow-up period.

[3] SHRs and 95% confidence intervals (95% CI) were estimated using the Fine-Gray competing risk models, treating death as a competing event for disability.

Supplemental Table S4. Multivariable-adjusted Odds Ratios of ADL and IADL Progression by Overall LE8 Score and Individual LE8 Component among Disability-free Participants at Baseline

LE8 score or components		Odds Ratios (95% CI)				
		ADL Disability (Sex-stratified)		p-value	IADL Disability	p-value
Overall LE8 Score		F	0.82 (0.66, 1.02)	0.072	0.76 (0.66, 0.88)	<0.001
		M	0.67 (0.49, 0.90)	0.009		
Moderate/High vs Low		F	0.50 (0.30, 0.85)	0.010	0.43 (0.30, 0.63)	<0.001
		M	0.43 (0.22, 0.84)	0.013		
Health Behavior Score		F	0.78 (0.62, 0.98)	0.031	0.85 (0.74, 0.97)	0.016
		M	0.82 (0.64, 1.05)	0.108		
Health Factor Score		F	0.84 (0.67, 1.06)	0.137	0.83 (0.72, 0.96)	0.010
		M	0.78 (0.51, 0.90)	0.083		
Health Behaviors	Diet	F	0.92 (0.74, 1.14)	0.448	0.96 (0.85, 1.10)	0.593
		M	1.01 (0.76, 1.35)	0.922		
	Physical Activity	F	0.57 (0.44, 0.75)	<0.001	0.62 (0.53, 0.72)	<0.001
		M	0.54 (0.40, 0.73)	<0.001		
	Smoking	F	0.98 (0.75, 1.27)	0.855	0.99 (0.87, 1.14)	0.911
		M	0.96 (0.74, 1.26)	0.789		
	Sleep	F	1.14 (0.93, 1.39)	0.200	0.90 (0.79, 1.03)	0.127
		M	0.89 (0.68, 1.18)	0.419		
Health Factors	BMI	F	0.89 (0.72, 1.11)	0.312	0.85 (0.75, 0.98)	0.022
		M	0.96 (0.73, 1.27)	0.778		
	Cholesterol	F	1.00 (0.80, 1.26)	0.991	1.02 (0.89, 1.16)	0.800
		M	0.97 (0.72, 1.29)	0.829		
	Blood Glucose	F	0.87 (0.70, 1.09)	0.233	0.90 (0.78, 1.03)	0.139
		M	0.88 (0.66, 1.16)	0.356		
	Blood Pressure	F	0.83 (0.66, 1.05)	0.129	0.83 (0.73, 0.95)	0.006
		M	0.62 (0.42, 0.91)	0.014		

[1] This table presents the odds ratios from GEE models with unstructured covariance that adjusted for participants' age, sex, study site, years of education, baseline cognitive status, baseline depression status, and presence of chronic disease at baseline.

Supplemental Table S5. Multivariable-adjusted Odds Ratios of ADL and IADL Progression by Overall LE8 Score (Per 1 SD) with Inverse Probability Weights (IPW)[1]

LE8 score or components	Odds Ratios (95% CI)				
	ADL Disability (Sex-stratified)		p-value	IADL Disability	p-value
Overall LE8 Score	F	0.88 (0.75, 1.04)	0.124	0.81 (0.73, 0.90)	<0.001
	M	0.62 (0.47, 0.81)	<0.001		
Moderate/High vs Low	F	0.63 (0.43, 0.92)	0.018	0.53 (0.40, 0.69)	<0.001
	M	0.38 (0.22, 0.65)	<0.001		
Health Behaviors	F	0.86 (0.71, 1.03)	0.091	0.88 (0.80, 0.97)	0.010
	M	0.80 (0.65, 0.98)	0.028		
Health Factors	F	0.90 (0.76, 1.07)	0.228	0.87 (0.78, 0.96)	0.008
	M	0.70 (0.54, 0.91)	0.007		

[1] This table presents odds ratios from GEE models with stabilized IPW to account for potential bias due to missing data. The GEE models were adjusted for participants' age, sex, study site, years of education, baseline cognitive status, baseline depression status, and presence of chronic disease at baseline.
